# Supplementary material for: Design, Synthesis, and Biological Evaluation of N-Acylhydrazones and Their Activity Against Leishmania amazonensis Promastigotes
Source: Microorganisms. 2025 Jul 2;13(7):1563. doi: 10.3390/microorganisms13071563 (PMC12298860; doi:10.3390/microorganisms13071563)
Supplement: Supplementary file 1 [file microorganisms-13-01563-s001.zip › HPLC amostras-20241114T144635Z-001.zip!/HPLC amostras/HPLC composto 14 - PMM 105 - 17.pdf]

Farmanguinhos - Fiocruz
Serviço de Métodos Analíticos

PMM 105 - 17.lcd
2018 - Sintese 1 - João Mafra - 2-5/2-74-4 - PMM 105 - 17.lcd
21/06/2018, 17:02:15

Sample Name : PMM 105 - 17
Sample ID : 118741
Sample Full Path : 2018 - Sintese 1 - João Mafra - 2-5/2-74-4 - PMM 105 - 17.lcd
Data Filename : PMM 105 - 17.lcd
Method Filename : Nitroimidazol.lcm
Batch File : 2018 - Sintese 1 - João Mafra - CLAE-10 - CLAE 10_2018 _0619.lcb
Vial # : 1-39 Sample Type : Unknown
Injection Volume : 10 uL
Date Acquired : 21/06/2018 17:02:44 Acquired by : Marcio Soares
Date Processed : 27/06/2018 08:55:59 Processed by : Marcio Soares
Data Comment : Método: Nitroimidazol

Preparo da Amostra: 1,13 mg foram adicionados 3 mL de metanol, agitada em vortex e levada ao ultrassom por 3 min. Posteriormente filtrada em membrana PVDF de 0,22µm e injetada.

Method

<<Header>>

<<Pump>>
Total Flow : 1,0000 mL/min
Pump A : LC-20ADXR

<<Mobile Phase Name>>
Pump A Mobile Phase A : Água
Pump A Mobile Phase C : Acetonitrila

<<LC Time Program>>

Time	Module	Command	Value	C
0,01	Pumps	Solvent C Conc.	20	
0,01	Pumps	Total Pump A Flow	1	
5,00	Pumps	Solvent C Conc.	20	
22,00	Pumps	Solvent C Conc.	90	
25,00	Pumps	Solvent C Conc.	90	
33,00	Pumps	Solvent C Conc.	20	
39,00	Pumps	Solvent C Conc.	20	
39,01	Controller	Stop		

<<Data Acquisition>>
LC Stop Time : 39,01 min

<<Oven>>
Oven Temperature : 30 C

Instrumentation

<<Instrument>>

<<Pump A>>
Pump A Type : LC-20ADXR

<<System Controller>>
SCL Type : CBM-20A

<<Autosampler>>
Autosampler : SIL-30AC

<<Oven>>
Oven Type : CTO-20AC

<<Column>>
Column Name : Supelcosil LC- 8 - 5 µm
Column ID : CL 0173
Length : 150 mm
Internal Diameter : 4,6 mm
Max Number of Injections : 9999
Expiration Date : 28/04/2015
Max Temperature : 80,0 C
Installation Date : 28/04/2030

<<PDA>>
Detector Name : PDA
Detector Type : SPD-M20A

<<Degasser>>
Degasser : DGU-20A5R

Farmanguinhos - Fiocruz
Serviço de Métodos Analíticos

PMM 105 - 17.lcd
2018 - Sintese 1 - João Mafra - 2-5/2-74-4 - PMM 105 - 17.lcd
21/06/2018, 17:02:15

Chromatogram

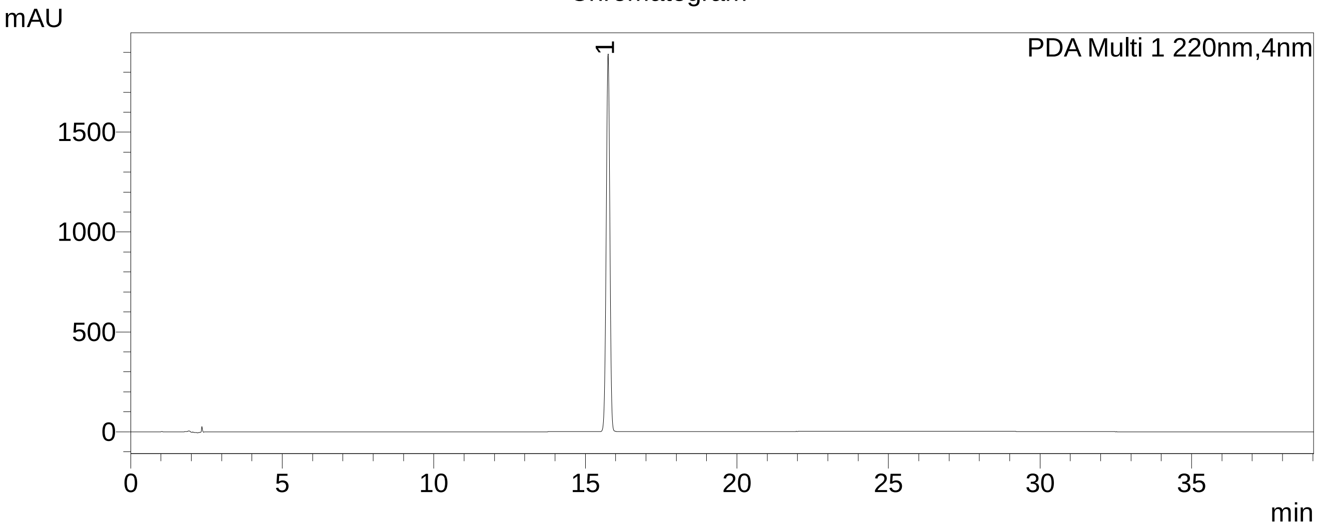

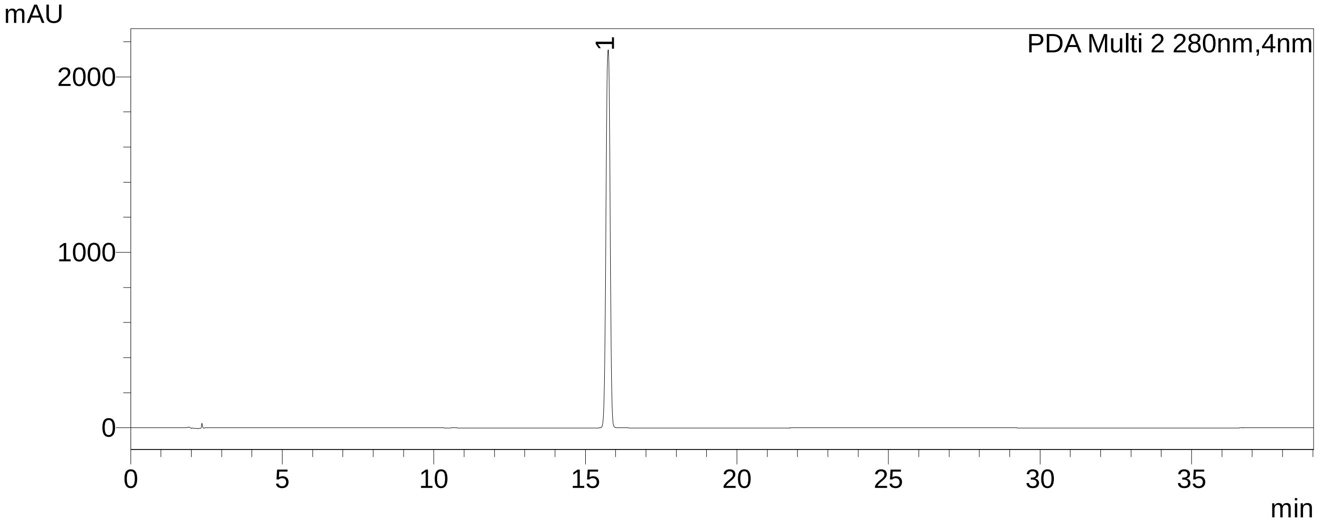

Peak Table

PDA Ch1 220nm

Peak#	Ret. Time	Name	Area	Area%	Theoretical Plates/meter(USP)	Tailing Factor	Resolution(USP)	Capacity Factor(k')
1	15,75		15823282	100,0	475264	0,972	--	--
Total			15823282	100,0				

PDA Ch2 280nm

Peak#	Ret. Time	Name	Area	Area%	Theoretical Plates/meter(USP)	Tailing Factor	Resolution(USP)	Capacity Factor(k')
1	15,75		20419762	100,0	424982	0,963	--	--
Total			20419762	100,0				
